# Insomnia and Migraine: A Missed Call?

**DOI:** 10.3390/clockssleep6010006

**Published:** 2024-02-05

**Authors:** Angelo Torrente, Lavinia Vassallo, Paolo Alonge, Laura Pilati, Andrea Gagliardo, Davide Ventimiglia, Antonino Lupica, Vincenzo Di Stefano, Cecilia Camarda, Filippo Brighina

**Affiliations:** 1 Department of Biomedicine, Neurosciences and Advanced Diagnostic (Bi.N.D.), University of Palermo, 90127 Palermo, Italy; angelo.torrente@unipa.it (A.T.); lavinia.v1994@gmail.com (L.V.); laura.pilati.91@gmail.com (L.P.); andrigl@gmail.com (A.G.); davideventimiglia211@gmail.com (D.V.); antlupica@gmail.com (A.L.); vincenzo19689@gmail.com (V.D.S.); cecilia.camarda@unipa.it (C.C.); filippobrighina@gmail.com (F.B.); 2 Neurology and Stroke Unit, P.O. “S. Antonio Abate”, 91016 Trapani, Italy; 3 Clinical Neurophysiology Unit, Sleep Lab, “Clinical Course”, 90143 Palermo, Italy

**Keywords:** migraine, sleep, insomnia, insomnia severity index, headache, patient-related outcome measures

## Abstract

Migraine is one of the most prevalent and disabling neurological conditions, presenting episodes of throbbing headache that limit activities of daily living. Several factors may influence migraine frequency, such as lifestyle or alcohol consumption. Among the most recognised ones, sleep plays a biunivocal role, since poor sleep quality may worsen migraine frequency, and a high migraine frequency may affect sleep quality. In this paper, the authors evaluate the relationship between migraine and insomnia by exploring a cohort of patients affected by episodic or chronic migraine. To do so, a phone interview was performed, asking patients about their migraine frequency and mean pain intensity, in addition to the questions of the Insomnia Severity Index. The last one explores several symptoms impairing sleep that focus on insomnia. Patients complaining of insomnia showed an increased migraine frequency, and a weak but significant correlation was found between headache days per month and insomnia scores. Such results were particularly evident in patients affected by chronic migraine. Such results suggest how insomnia, in the presented data, seems to be associated with migraine frequency but not with pain intensity.

## 1. Introduction

Migraine is a neurological disorder characterised by throbbing, unilateral and severe headache episodes accompanied by symptoms like nausea, photophobia and phonophobia [1]. The pain is usually of moderate to severe intensity and may last even up to 3 consecutive days, affecting patients’ daily work and leisure activities. Its disabling nature and its high prevalence make it one of the most considered neurological disorders. Indeed, 14.7% of the adult population suffers from migraine, with a significant reduction in the quality of life and high management costs, which are both direct and indirect (e.g., work absenteeism or presenteeism) [2,3]. Moreover, in adulthood, there is a higher prevalence in the female sex, which can be even more than trifold compared to males [4].

Based on migraine frequency, the International Headache Society in the most recent International Classification of Headache Disorders, 3rd edition (ICHD-3), distinguishes between chronic (CM—i.e., if a patient complains for more than 3 months of 15 or more days of headache per month and at least 8 of which show migraine characteristics or lead to acute drug intake) and episodic migraine (EM—i.e., when the patient does not meet the above-mentioned criteria) [5]. 

Despite in the general knowledge migraine being considered a phasic disease, patients show symptoms before (i.e., prodromal or preictal phase—usually up to 48 h), during (i.e., ictal phase—usually from 4 to 72 h), after (i.e., postdromal or postictal phase—up to 48 h) and between headache episodes (i.e., interictal phase), configuring the so-called migraine cycle [6]. In some patients, another phase could be included, which is the aura one, characterised by the onset of progressive and transient focal neurological symptoms lasting usually up to one hour each and presenting typically immediately before or during a migraine attack. This subpopulation of patients affected by migraine with aura (MwA) is slightly different from the one without aura (MwoA) as it shows even more marked pathophysiological changes [7]. The cyclic aspect of the disease (giving dignity and relevance even to the interictal phase) may even explain several cognitive (e.g., subjective memory impairment) and psychological symptoms (e.g., anxiety or depression) that migraine patients complain of [8,9]. 

Migraine shows a strong link with a multitude of both physiological (e.g., lifestyle, physical activity) and pathological factors (e.g., stress, anxiety, depression, fibromyalgia, obesity) [9,10]. One of the most recognised and yet not completely understood is the bilateral relationship between migraine and sleep, since on one hand, a poor sleep quality may worsen migraine frequency and, on the other hand, an increase in migraine frequency or overnight onset of attacks may affect sleep quality [11,12,13,14]. As a result, migraineurs show a reduction in sleep quality and efficiency [15]. Sleep is a key factor in maintaining the functions not only of the central nervous system but also of other systems, and sleep disturbances are indeed related to an increase in total mortality, cardiovascular diseases, diabetes, respiratory disorders, obesity, and perceived poor health [16]. Stressing the importance of the link with migraine, sleep has even been demonstrated to influence cortical excitability throughout the migraine cycle: recent evidence proved that a night of 4 h of sleep in the preictal phase leads to the dysfunction of the cortical GABAergic inhibition and that during the pain phase, sleep itself seems to be a key element to maintain a normal neurological function after the migraine attack [17]. As a matter of fact, during a migraine attack, several patients report finding relief simply by going to sleep or taking a nap. On the other hand, the lack of sleep may affect migraine patients due to a rise in adenosine levels, which is a precipitating factor for migraine attacks. In addition, sleep deprivation may even provoke an increased susceptibility to cortical spreading depression because of a rise in cortical glutamate levels [18]. Another link between migraine and sleep may rely on the overlapping transmitters involved in their physiology, such as orexin, dopamine, serotonin, and norepinephrine. These ones regulate sleep but are also involved in pain transmission, processing, and modulation [18].

Insomnia is the most prevalent sleep disorder and may manifest in difficulty in starting or maintaining sleep, early awakenings, or an overall reported bad quality of sleep associated with daily living impairment [19]. Poor sleep quality and insomnia may be associated with several conditions such as chronic pain syndromes, but even psychological ones, such as anxiety or depression [20]. Despite the last ones remaining among the most recognised factors influencing sleep quality in the general population [21], some evidence highlights how this may not be entirely true in the migraineurs population, and other specific factors may play a role [15]. Insomnia and migraine may find a common physiopathological role in hypothalamus and brainstem dysfunctions. These structures are indeed involved in the sleep–wake cycle and pain modulation [14]. Furthermore, looking at recent global events, data showed how even the social distancing due to the COVID-19 pandemic increased insomnia symptoms in migraineurs [22]. 

Given the high prevalence of both migraine and insomnia, it may be easy to conclude that many individuals may suffer from the two of them at the same time; however, this link is not merely epidemiological, but these conditions are known even to affect each other [11]. The main objective of the present study is to investigate the correlation between migraine features and insomnia symptoms to increase our knowledge of this complex bilateral relationship.

## 2. Results

### 2.1. Population and Migraine Features

We interviewed 156 patients with a mean age of 43 ± 12 years, among whom 132 (84.6%) were women. Among the total population, 62 (39.7%) patients were affected by CM, and overall, 45 (28.8%) showed symptoms of aura on some occasions (MwA). The demographic and clinical characteristics of the population are found in Table 1. 

### 2.2. Insomnia Symptoms and Correlation with Migraine Features

Analysing the Insomnia Severity Index (ISI) questionnaire, the overall population showed an ISI total score of 8.3 ± 6.8; see Table 2. Based on the ISI score, 49.4% of patients fell in the ISI category “absence of insomnia”, while 50.6% fell into one of any insomnia categories (i.e., from “subthreshold insomnia” and above). Comparing the two groups (i.e., no insomnia vs. any insomnia), we found that the first ones showed a reduced number of headache days (no insomnia 9.7 ± 7.5, any insomnia 13.5 ± 8.7, *p* = 0.004), while there were no significant differences in pain intensity (no insomnia 6.9 ± 1.9, any insomnia 7.3 ± 1.6, *p* = 0.193). 

Overall, the difficulty in falling asleep score (item 1a) and in maintaining sleep (item 1b) showed a significant correlation with headache frequency (*p* < 0.05). In addition, item 5 and the ISI total score showed a similar significant but weaker correlation (see Table 2). On the other hand, the other items did not show any significant correlation. Similarly, insomnia scores were not significantly correlated with the intensity of pain.

#### 2.2.1. Analysis of Chronic vs. Episodic Migraineurs Subpopulations

Table 3 shows the distribution of the ISI insomnia categories among the overall, CM, and EM populations. EM patients showed more often an absence of insomnia, while the percentage of subthreshold insomnia is more represented among CM ones. 

When comparing the results of the ISI questionnaire raw scores between the two groups, we found that CM patients showed slightly higher scores in the ISI items 1a (CM 1.6 ± 1.5 vs. EM 1.0 ± 1.3, *p* = 0.011) and 1b (CM 1.4 ± 1.4 vs. EM 0.9 ± 1.2, *p* = 0.024). The complete results are listed in Table 4.

The correlation analysis performed on CM patients showed a slightly stronger correlation between headache days and item 1b (r = 0.328, *p* = 0.009) and with item 1c (r = 0.310, *p* = 0.014), while EM patients’ scores did not show any significant correlation (see Table 5 and Table 6). The distribution of ISI subclass diagnoses was different from the expected (chi-square *p* = 0.043). In addition, the presence of any ISI category (subthreshold, moderate, or severe) vs. absence of insomnia was compared between CM and EM and revealed a significant difference at the chi-square test (CM showing any insomnia in 62.9%, while there was no insomnia in 37.1%; EM showing any insomnia in 42.6%, while there was no insomnia in 57.4%, *p* = 0.032). 

#### 2.2.2. Comparison of Migraine with or without Aura Subpopulations

There were no significant differences between MwA and MwoA patients regarding headache days per month (MwA 11.2 ± 8.4 vs. MwoA 11.8 ± 8.3, *p* = 0.669) or pain intensity (MwA 7.4 ± 1.7 vs. MwoA 7.0 ± 1.8, *p* = 0.220). Nevertheless, since the two subtypes of migraine may show different features, we decided to compare the ISI scores results. The analysis showed a slight increase in item 1c scores in the MwA group compared to the MwoA one (see Table 7).

#### 2.2.3. Analysis of Patients Reporting vs. Non-Reporting History of Anxiety Subpopulations

The frequency of the ISI categories among the different populations based on the presence of self-reported anxiety is shown in Table 8. 

Interestingly, no significant correlation with headache days was present when analysing the ISI scores of patients complaining of anxiety (see Table 9), while it was still weakly present in non-anxious patients for item 1a (r = 0.241, *p* = 0.030) and item 1b (r = 0.320, *p* = 0.004, see Table 10). Even here, the distribution of ISI subclass diagnoses was different from the expected (chi-square *p* = 0.002). 

## 3. Discussion

The present study aimed to add insight into the connection between migraine and insomnia, correlating the results of an insomnia index with headache frequency and intensity reported by patients. Prospective studies have already demonstrated the tight biunivocal relationship between migraine and insomnia. So, patients with insomnia show a higher risk of developing migraine than the ones without it, and migraine patients show a higher risk of insomnia compared to headache-free subjects [23,24,25]. In our cohort, patients presenting any grade of insomnia showed an increased number of headache days per month. When trying to evaluate which insomnia aspect would have been more influenced by headache frequency, considering the overall population, we found a weak yet statistically significant association between the difficulty in falling asleep and staying asleep with the number of headache days per month. The first aspect may be related to the difficulties in falling asleep in a patient suffering from long-lasting pain, which may not cease rapidly even with a correct acute medication use. The second relation, instead, may be due to the frequent onset of migraine attacks even during sleep, as it happens in cluster headache [26]. On the other hand, we can speculate that the two types of insomnia associated with an increase in migraine frequency (and that may be looked for and addressed during the visits at the headache outpatient clinics) are the initial (i.e., falling asleep) and the middle (i.e., maintenance) insomnia. Moreover, a weak correlation was even found between headache frequency and the perceived distress induced by insomnia, thus strengthening the link between psychological symptoms and migraine frequency. Instead, the other insomnia items did not correlate with headache days per month except for the overall score of the index. 

There are reports in the literature showing that migraineurs with insomnia present on average a higher intensity of attacks compared to the ones not complaining of insomnia [27]. Nevertheless, in our cohort, we were not able to detect any significant difference in pain intensity between the subjects showing any grade of insomnia versus the ones without insomnia. Accordingly, none of the ISI items showed any significant correlation with the intensity of migraine pain. Such a difference with the literature may be due to the relatively low numerosity of our sample or to a different cut-off for the definition of insomnia based on ISI scores. We classified with “any insomnia” the patients showing ISI scores able to classify them to the group “subthreshold insomnia” and above (i.e., an ISI score ≥ 8), while the above-cited group chose a higher cut-off (ISI score ≥ 10) [27]. This last choice may have led the latter authors to select insomnia patients with worse migraine features. Based on our results, a worsening of insomnia should not be related to a worsening of headache intensity. Moreover, these last results may indicate how migraine influence on sleep is not dependent on the intensity of the pain (which is always, by definition, generally moderate to high) but rather only on its frequency. 

The higher the migraine attacks frequency, the more insomnia symptoms there are [14]. To confirm such data, when analysing the subpopulations of patients, CM (i.e., the more severe disease, with a higher number of headache days per month) showed a slightly stronger correlation of headache frequency with sleep maintenance and early awakening. On the other hand, EM (i.e., fewer headache days per month) patients did not show any significant correlation. With a partially different result, looking just at the ISI scores, CM patients showed slightly more difficulties falling asleep and maintaining sleep than EM. This last result could indicate that the increased frequency of headaches during the day may make falling asleep more difficult in CM patients. The connection between poor sleep quality and migraine has been known for a long time and concerns both subjective and objective sleep parameters. Data show that both poor sleep quality detected via patient-oriented outcome measures (PROMs) questionnaires and a reduction in REM sleep evaluated using polysomnography may act as exacerbating factors for migraine attacks and chronification (i.e., the progression from EM to CM) [11]. Furthermore, it has been demonstrated that CM patients show poor sleep quality, which nevertheless may be improved by preventive therapies [28,29].

Surprisingly, we found a slight difference even when comparing MwA with MwoA, since the former complained of early awakenings slightly more than the latter. This last result may indicate that MwA may present an increased onset of migraine attacks in the last part of the night or the early morning. It is indeed not unusual for migraine attacks to follow a circadian presentation [30]. Migraine itself can deeply worsen the quality of sleep, since a great percentage of migraine attacks begin during nocturnal sleep (mostly between 4 and 9 a.m.) as often spontaneously reported by patients [31]. Interestingly, not only can a reduced amount of sleep trigger migraine attacks, but also an excessive quantity of sleep, as well as a delayed to-sleep and wake-up time, can bring the same consequence: this is one of the mechanisms believed to contribute to the so-called “weekend migraine”, which is an annoying phenomenon consisting of the onset of migraine attacks during weekends or days off [32]. Indeed, different sleep habits, such as the tendency to go to sleep or to wake up at a certain time (i.e., chronotype) may influence migraine frequency and onset [33].

Furthermore, when considering patients reporting anxiety, no significant correlation was found between ISI items and headache features in our cohort. These results could mean that in such patients, the main cause of insomnia may be addressed to the psychological symptom and not to headache. 

In addition, it is worth mentioning that migraine is associated with sleep disturbances as frequent comorbidities: a relevant example is the restless leg syndrome, which is characterised by abnormal leg sensations while resting in bed during nocturnal sleep, causing insomnia and interfering with daily living [34]; it is demonstrated that the co-occurrence of both disorders (i.e., migraine and RLS) worsens their severity with a bidirectional relationship [14].

A considerable part of sleep disturbances, especially in adolescents and young adults during their studies, is affected by unhealthy habits like taking naps lasting more than 1 h, going to sleep and waking from sleep at different times from day to day, consuming caffeine in the late afternoon or early evening, using electronic devices right before bedtime, late night studying, an uncomfortable room or bed and roommates with different sleep schedules [35]. It appears therefore crucial that an effort in educating people is needed, especially for patients affected by migraine or sleep disturbances or both, to acquire healthy lifestyle habits that are likely to improve their conditions.

The presented study shows some strengths: the phone interview was administered by a headache expert neurologist, insomnia was assessed using a validated questionnaire, and the patients based their answers on a headache diary, as they were already trained to, avoiding the risk of recall bias. However, the study presents several limitations, too. First, the sample does not include many patients, so future studies with wider populations are needed to obtain more robust results. According to migraine epidemiology, the overall population was mainly composed of females (84.6%), and the limited number of males would not have provided robust results for a gender-based subgroup analysis. So, we could not assess any sex influence on the results. In addition, the analyses did not include a specific anxiety or depression scale, whose implementation would have been useful to measure the influence of these factors on insomnia. Anxiety and depression are great predictors of future insomnia, and insomnia itself is a predictor of future anxiety and depression [36]; these conditions likely have an even greater impact on insomnia than headache days per month, whose influence it is, according to our data, weak. It is therefore clear the great interrelationship between such conditions, making clear the remarkable clinical implications of this link and acting as a stimulus for further research and data expansion. Other data that could have been added to give more insight into sleep could have been the study of patients’ chronotypes. Moreover, a limitation could be represented by the use of a questionnaire, which is patient-dependent, instead of an instrumental tool such as actigraphy or polysomnography. A future direction for a more reliable measure to establish migraine attacks’ influence on insomnia could be to include its assessment in a day-by-day fashion in headache diaries. 

## 4. Materials and Methods

### 4.1. Patients Collection and Migraine Features

A phone interview with migraine patients referred at the Headache Centre of the University Hospital “Paolo Giaccone” of Palermo, Italy, was conducted by a headache expert neurologist. The consecutive patients referred to the centre who were willing to participate were informed about all the details of the research study, being reassured that all the information provided would have been analysed and published anonymously. After having the time to think about it and to ask the neurologist any questions about the research, patients willing to participate gave their consent to the study. The included patients were affected by EM or CM with or without aura according to the ICHD-3 criteria [5] and were older than 18 years old. The authors decided to exclude all the patients who had a history of overt sleep or psychiatric disorder as well as the ones who had been taking any medication with a possible influence on sleep (e.g., benzodiazepines). Moreover, we excluded patients affected by other neurological disorders but migraine or any other chronic pain syndrome (e.g., musculoskeletal, rheumatological, fibromyalgia, sensory neuropathy) that could have influenced sleep quality. Patients were asked to report the number of headache days per month (on average of the last three months) and the mean pain intensity on a numeric rating scale (NRS—from 0 to 10). All patients were accustomed to collecting the data requested, since they were the usual ones requested during follow-up evaluations at the outpatient clinic. 

### 4.2. Insomnia Study

The Italian version of the Insomnia Severity Index (ISI) questionnaire was administered via telephone. This one evaluates the severity of sleep disturbances associated with insomnia exploring 5 different items: the first one evaluates the presence and the intensity of difficulties falling asleep, maintaining sleep, and the entity of early morning awakenings (i.e., subitems 1a, 1b, and 1c, respectively); the second item assesses the satisfaction level with the patient’s current sleep pattern; the third one considers the perceived interference with daily living; the fourth one investigates the external noticeability of such impairment; finally, the fifth item evaluates the distress caused by insomnia. Each question score can go from 0 (no disturbance) to 4 (very severe disturbance) so the final score of the questionnaire ranges from 0 to 28 with higher scores meaning more insomnia symptoms [37]. The results of the score may suggest absence (0–7), subthreshold (8–14), moderate (15–21), or severe insomnia (22–28).

### 4.3. Subjective Anxiety Assessment

Due to the strong correlation between anxiety and insomnia, investigators chose to have a subjective measure for the presence or absence of the former among the studied patients. For this reason, we simply asked patients whether they had the perception to be a generally anxious person or to suffer from anxiety. 

### 4.4. Statistical Analysis

Qualitative variables were presented as percentages, while quantitative ones were presented as mean ± standard deviation (SD); the correlation analyses were performed using Spearman’s rank coefficient. Subanalyses were performed even considering the subpopulations (EM vs. CM, and anxious vs. non-anxious patients). Quantitative variables have been analysed using a Mann–Whitney U test, while qualitative ones were analysed using the chi-square McNemar’s paired test. Analyses were performed using SPSS v. 26 software by I.B.M., Armonk, NY, USA. 

## 5. Conclusions

The present study shows how patients suffering from migraine and insomnia show an increased headache frequency compared to the ones without insomnia. Accordingly, a weak though present relation can be observed between headache frequency and insomnia symptoms in patients affected by migraine. Despite the weak influence, it appears to be more pronounced in patients affected by CM. Moreover, slight differences could be found even when comparing MwA with MwoA patients. Despite different findings in the literature, in the presented cohort, no influence between referred headache intensity and insomnia has been detected. Lastly, the presence of a self-recognised anxious phenotype may reduce the influence between headache frequency and insomnia, so in those patients, anxiety may represent the most influencing factor. 

Finally, we can say that there is a high prevalence of insomnia symptoms among migraineurs, so insomnia may be further assessed during headache outpatient clinics. Based on the reported biunivocal relation, treating insomnia in the first place may even improve migraine without specific prophylaxes as well as treating migraine may improve patients’ reported insomnia.

## Figures and Tables

**Table 1:** Summary of patients’ clinical and demographical features. CM: chronic migraine; EM: episodic migraine; SD: standard deviation.

Patients’ Characteristics	
Total, n (%)	156 (100%)
Age (±SD)	43 ± 12
Sex, n (%)	Female 132 (84.6%) Male 24 (15.4%)
Headache days/month (±SD)	11.7 ± 8.3
Pain intensity (±SD)	7.1 ± 1.7
Diagnosis, n (%)	CM 62 (39.7%) EM 94 (60.3%)
MwA, n (%)	45 (28.8%)
History of anxiety, n (%)	Yes 75 (48.1%) No 81 (51.9%)

Abbreviations: CM = chronic migraine; EM = episodic migraine; ISI = Insomnia Severity Index; MwA = migraine with aura; SD = standard deviation.

**Table 2:** General population correlation analysis among the ISI score, the number of headache days per month and pain intensity.

ISI Item General Population	Score Mean ± SD	Spearman’s Correlation Analysis	
		Headache Days/Month r (*p* Value)	Pain Intensity r (*p* Value)
1a	1.2 ± 1.4	0.212 (0.008) **	0.079 (0.330)
1b	1.1 ± 1.3	0.215 (0.007) **	0.080 (0.321)
1c	0.7 ± 1.1	0.017 (0.830)	−0.007 (0.934)
2	1.7 ± 1.4	0.019 (0.811)	0.145 (0.070)
3	1.4 ± 1.4	0.059 (0.461)	0.037 (0.644)
4	1.1 ± 1.3	0.110 (0.170)	0.040 (0.622)
5	1.1 ± 1.3	0.182 (0.023) *	0.107 (0.186)
Total score	8.3 ± 6.8	0.165 (0.039) *	0.109 (0.174)

Items: 1a = difficulties in falling asleep, 1b = difficulties in maintaining sleep, 1c = entity of early morning awakenings, 1a = difficulties in falling asleep, 2 = satisfaction level with the current sleep pattern, 3 = perceived interference with daily living, 4 = external noticeability of sleep impairment, and 5 = distress caused by insomnia. Abbreviations: ISI = Insomnia Severity Index; SD = standard deviation. * *p* < 0.05; ** *p* < 0.01.

**Table 3:** Frequency of the ISI categories among the different populations based on headache frequency.

ISI Insomnia Category	Overall (156)	CM (62)	EM (94)
absence (0–7)	77 (49.4%)	23 (37.1%)	54 (57.4%)
subthreshold (8–14)	44 (28.2%)	23 (37.1%)	21 (22.3%)
moderate (15–21)	29 (18.6%)	14 (22.6%)	15 (16%)
severe (22–28)	6 (3.8%)	2 (3.2%)	4 (4.3%)

Abbreviations: CM = chronic migraine; EM = episodic migraine; ISI = Insomnia Severity Index; SD = standard deviation.

**Table 4:** Chronic vs. episodic migraineurs ISI scores comparison.

ISI Item	CM Mean ± SD	EM Mean ± SD	*p* Value
1a	1.6 ± 1.5	1.0 ± 1.3	0.011 *
1b	1.4 ± 1.4	0.9 ± 1.2	0.024 *
1c	0.6 ± 1.0	0.8 ± 1.1	0.442
2	1.8 ± 1.4	1.7 ± 1.4	0.677
3	1.5 ± 1.4	1.3 ± 1.3	0.439
4	1.2 ± 1.3	1.0 ± 1.3	0.253
5	1.3 ± 1.3	1.0 ± 1.3	0.095
Total score	9.4 ± 6.8	7.5 ± 6.8	0.100

Items: 1a = difficulties in falling asleep, 1b = difficulties in maintaining sleep, 1c = entity of early morning awakenings, 1a = difficulties in falling asleep, 2 = satisfaction level with the current sleep pattern, 3 = perceived interference with daily living, 4 = external noticeability of sleep impairment, and 5 = distress caused by insomnia. Abbreviations: CM = chronic migraine; ISI = Insomnia Severity Index. * *p* < 0.05.

**Table 5:** CM correlation analysis among the ISI score, the number of headache days per month and pain intensity.

ISI Item CM	Score	Spearman’s Correlation Analysis	
		Headache Days/Month r (*p* Value)	Pain Intensity r (*p* Value)
1a	1.6 ± 1.5	0.021 (0.869)	0.058 (0.655)
1b	1.4 ± 1.4	0.328 (0.009) **	−0.066 (0.609)
1c	0.6 ± 1.0	0.310 (0.014) *	−0.054 (0.674)
2	1.8 ± 1.4	0.239 (0.061)	0.082 (0.525)
3	1.5 ± 1.4	0.077 (0.551)	0.021 (0.873)
4	1.2 ± 1.3	−0.014 (0.913)	−0.022 (0.866)
5	1.3 ± 1.3	0.094 (0.470)	0.160 (0.215)
Total score	9.4 ± 6.8	0.187 (0.145) *	0.034 (0.793)

Items: 1a = difficulties in falling asleep, 1b = difficulties in maintaining sleep, 1c = entity of early morning awakenings, 1a = difficulties in falling asleep, 2 = satisfaction level with the current sleep pattern, 3 = perceived interference with daily living, 4 = external noticeability of sleep impairment, and 5 = distress caused by insomnia. Abbreviations: CM = chronic migraine; ISI = Insomnia Severity Index. * *p* < 0.05; ** *p* < 0.01.

**Table 6:** EM correlation analysis among the ISI score, the number of headache days per month and pain intensity.

ISI Item EM	Score	Spearman’s Correlation Analysis	
		Headache Days/Month r (*p* Value)	Pain Intensity r (*p* Value)
1a	1.0 ± 1.2	0.089 (0.394)	0.059 (0.571)
1b	0.9 ± 1.2	0.025 (0.810)	0.121 (0.245)
1c	0.8 ± 1.1	0.065 (0.532)	0.047 (0.656)
2	1.7 ± 1.4	−0.119 (0.255)	0.167 (0.107)
3	1.3 ± 1.3	−0.020 (0.850)	0.035 (0.737)
4	1.0 ± 1.3	0.096 (0.356)	0.068 (0.513)
5	1.0 ± 1.3	0.148 (0.154)	0.053 (0.614)
Total score	7.6 ± 6.8	0.034 (0.743)	0.108 (0.302)

Items: 1a = difficulties in falling asleep, 1b = difficulties in maintaining sleep, 1c = entity of early morning awakenings, 1a = difficulties in falling asleep, 2 = satisfaction level with the current sleep pattern, 3 = perceived interference with daily living, 4 = external noticeability of sleep impairment, and 5 = distress caused by insomnia. Abbreviations: EM = episodic migraine; ISI = Insomnia Severity Index.

**Table 7:** Migraineurs with and without aura subpopulations ISI scores comparison.

ISI Item	MwA Mean ± SD	MwoA Mean ± SD	*p* Value
1a	1.3 ± 1.4	1.2 ± 1.4	0.758
1b	1.3 ± 1.4	1.0 ± 1.3	0.173
1c	1.1 ± 1.3	0.6 ± 1.0	0.026 *
2	1.6 ± 1.4	1.8 ± 1.4	0.491
3	1.5 ± 1.5	1.3 ± 1.3	0.482
4	1.2 ± 1.3	1.0 ± 1.3	0.335
5	1.4 ± 1.4	1.0 ± 1.3	0.096
Total score	9.4 ± 7.4	7.8 ± 6.6	0.183

Items: 1a = difficulties in falling asleep, 1b = difficulties in maintaining sleep, 1c = entity of early morning awakenings, 1a = difficulties in falling asleep, 2 = satisfaction level with the current sleep pattern, 3 = perceived interference with daily living, 4 = external noticeability of sleep impairment, and 5 = distress caused by insomnia. Abbreviations: ISI = Insomnia Severity Index; MwA = migraine with aura; MwoA = migraine without aura. * *p* < 0.05.

**Table 8:** Frequency of the ISI categories among the different populations based on the presence or absence of self-perceived anxiety.

ISI Insomnia Category	Overall (156)	Anxious (75)	Non-Anxious (81)
absence (0–7)	77 (49.4%)	27 (30.0%)	50 (61.7%)
subthreshold (8–14)	44 (28.2%)	23 (30.7%)	21 (25.9%)
moderate (15–21)	29 (18.6%)	22 (29.3%)	7 (8.6%)
severe (22–28)	6 (3.8%)	3 (4.0%)	3 (3.7%)

Abbreviations: ISI = Insomnia Severity Index.

**Table 9:** Correlation analysis among the ISI score, the number of headache days per month and pain intensity in patients with a reported history of anxiety.

ISI Item Anxious	Score	Spearman’s Correlation Analysis	
		Headache Days/Month r (*p* Value)	Pain Intensity r (*p* Value)
1a	1.6 ± 1.4	0.162 (0.165)	−0.040 (0.732)
1b	1.2 ± 1.4	0.077 (0.513)	0.084 (0.472)
1c	0.8 ± 1.2	−0.100 (0.393)	−0.021 (0.861)
2	2.1 ± 1.3	0.002 (0.988)	0.182 (0.119)
3	1.6 ± 1.4	−0.036 (0.759)	0.096 (0.412)
4	1.5 ± 1.3	0.037 (0.751)	−0.020 (0.865)
5	1.6 ± 1.4	0.115 (0.327)	0.018 (0.877)
Total score	10.4 ± 6.8	0.065 (0.580)	0.066 (0.576)

Items: 1a = difficulties in falling asleep, 1b = difficulties in maintaining sleep, 1c = entity of early morning awakenings, 1a = difficulties in falling asleep, 2 = satisfaction level with the current sleep pattern, 3 = perceived interference with daily living, 4 = external noticeability of sleep impairment, and 5 = distress caused by insomnia. Abbreviations: ISI = Insomnia Severity Index.

**Table 10:** Correlation analysis among the ISI score, the number of headache days per month and pain intensity in patients without a reported history of anxiety.

ISI Item Non-Anxious	Score	Spearman’s Correlation Analysis	
		Headache Days/Month r (*p* Value)	Pain Intensity r (*p* Value)
1a	0.8 ± 1.2	0.241 (0.030) *	0.075 (0.506)
1b	1.0 ± 1.3	0.320 (0.004) **	0.045 (0.690)
1c	0.7 ± 1.0	0.118 (0.295)	0.003 (0.976)
2	1.4 ± 1.4	−0.035 (0.759)	0.035 (0.759)
3	1.1 ± 1.3	0.081 (0.473)	−0.067 (0.555)
4	0.7 ± 1.2	0.101 (0.370)	−0.004 (0.972)
5	0.7 ± 1.1	0.202 (0.071)	0.116 (0.304)
Total score	6.4 ± 6.4	0.153 (0.172)	0.049 (0.665)

Items: 1a = difficulties in falling asleep, 1b = difficulties in maintaining sleep, 1c = entity of early morning awakenings, 1a = difficulties in falling asleep, 2 = satisfaction level with the current sleep pattern, 3 = perceived interference with daily living, 4 = external noticeability of sleep impairment, and 5 = distress caused by insomnia. Abbreviations: ISI = Insomnia Severity Index. * *p* < 0.05; ** *p* < 0.01.

## Data Availability

Permission will be given after reasonable request to the corresponding author and guaranteeing the proper citation of the paper.
